# White Matter Lesion Volumes on 3-T MRI in People With MS Who Had Followed a Diet and Lifestyle Program for More Than 10 Years

**DOI:** 10.1155/2024/8818934

**Published:** 2024-11-01

**Authors:** Mariaan Jaftha, Frances Robertson, Susan J. van Rensburg, Martin Kidd, Ronald van Toorn, Merlisa C. Kemp, Clint Johannes, Kelebogile E. Moremi, Lindiwe Whati, Maritha J. Kotze, Penelope Engel-Hills

**Affiliations:** ^1^Department of Medical Imaging and Therapeutic Sciences, Faculty of Health and Wellness Sciences, Cape Peninsula University of Technology, Bellville, Cape Town, South Africa; ^2^Cape University Body Imaging Centre, Faculty of Health Sciences, University of Cape Town, Cape Town, South Africa; ^3^Department of Human Biology, Faculty of Health Sciences, University of Cape Town Neuroscience Institute, University of Cape Town, Cape Town, South Africa; ^4^Division of Chemical Pathology, Department of Pathology, Faculty of Medicine and Health Sciences, Stellenbosch University, Cape Town, South Africa; ^5^Centre for Statistical Consultation, Department of Statistics and Actuarial Sciences, Stellenbosch University, Private Bag X1, Matieland 7602, Cape Town, South Africa; ^6^Department of Pediatrics and Child Health, Faculty of Medicine and Health Sciences, Stellenbosch University, Cape Town, South Africa; ^7^Medical Imaging, Department of Health and Care Professions, Faculty of Health and Life Sciences, University of Exeter, Exeter, UK; ^8^Department of Internal Medicine, Faculty of Medicine and Health Sciences, Stellenbosch University, Tygerberg 7500, Cape Town, South Africa; ^9^Division of Chemical Pathology, Department of Pathology, Faculty of Medicine and Health Sciences, Stellenbosch University, and National Health Laboratory Service (NHLS), Cape Town, South Africa; ^10^Gknowmix (Pty) Ltd., Bellville 7530, South Africa; ^11^Faculty of Health and Wellness Sciences, Cape Peninsula University of Technology, Cape Town, South Africa

**Keywords:** diet, lifestyle, MRI, multiple sclerosis, SAMSEG, white matter lesion volumes

## Abstract

**Background**: Cerebral white matter lesion (WML) formation in people with multiple sclerosis (pwMS) is linked to the death of myelin-producing oligodendrocytes. Current MS treatment strategies focus on limiting WML accumulation and disability. Using a pathology-supported genetic testing (PSGT) program, we identified specific risk factors for MS, categorized as *deficiencies* and *aggravators*. We developed a novel clinical methodology to mitigate these risk factors, including personalized lifestyle interventions and optimization of cerebral nutrients to prevent oligodendrocyte demise and promote remyelination.

**Objective**: To conduct a pilot case-control study over a 10-year period to ascertain whether the PSGT Program can reduce or prevent WML formation in pwMS.

**Methods**: MRI was performed at baseline as well as after an interval period of at least 10 years or longer in 22 pwMS. WML volumes were determined using Sequence Adaptive Multimodal SEGmentation (SAMSEG) software, part of FreeSurfer 7.2. Other variables included age at MRI, disease duration, disability status, and medication.

**Results**: PwMS (*n* = 13) who had followed the PSGT program for more than 10 years, had significantly smaller lesion volumes (mm^3^) compared to pwMS who did not adhere to the program (*n* = 9) (4950 ± 5303 vs. 17934 ± 11139; *p* = 0.002). WML volumes were significantly associated (*p* = 0.02) with disability (EDSS) but not with age (*p* = 0.350), disease duration (*p* = 0.709), or Interferon-*β* treatment (*p* = 0.70).

**Conclusion:** Dietary and lifestyle changes may lower the risk of developing cerebral WMLs in pwMS and potentially slow disease progression. Larger studies are required to confirm the effectiveness of such interventions in pwMS.

## 1. Introduction

**Figure 1:**
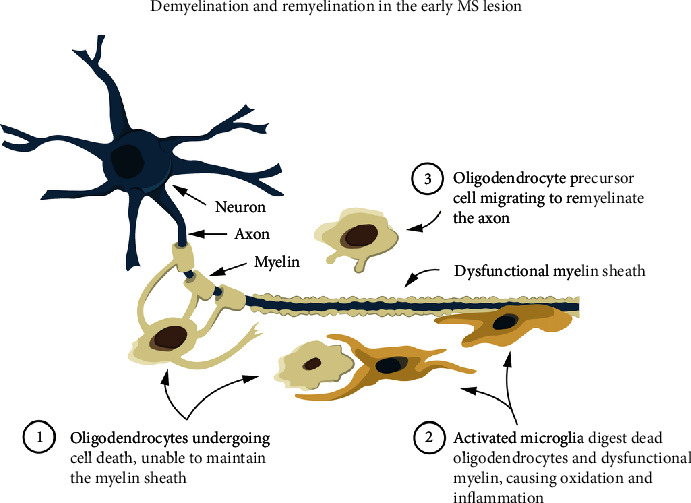
Demyelination and remyelination of the early MS lesion as revealed by neuropathological studies [1, 2]. Oligodendrocytes experience cell death and are unable to maintain the myelin sheath; then, activated microglia or monocyte-derived macrophages digest (scavenge) the dead oligodendrocytes and dysfunctional myelin without damaging the axons. Thereafter, oligodendrocyte precursor cells (OPCs) become the new oligodendrocytes and remyelinate the axons, restoring functionality.

Multiple sclerosis (MS), the most common inflammatory disorder of the central nervous system (CNS) affecting young adults, is associated with damage to the myelin sheaths surrounding the nerve axons in the CNS. This causes disruption of signal transmission within the CNS and from the CNS to the peripheral organs, resulting in disability (loss of function). Radiologically, this manifests as cerebral white matter lesions (WMLs) [1]. Neuropathological studies confirm that early WMLs are associated with the loss of myelin *secondary* to the death of myelin-producing oligodendrocytes [1]. The precise mechanism of cell death and phagocytosis of myelin are still unclear as both activated microglia (local macrophages) and monocyte–derived macrophages from the circulation remove dead oligodendrocytes and dysfunctional myelin without damaging the axons [2], while T-cells are rare and plasma cells (B-cells) are absent in these lesions [1–4]. Concomitantly, oligodendrocyte precursor cells (OPCs) migrate to the site of the lesion and remyelinate the axons [1, 2]. (Figure 1). OPCs are stem cells originating in the ventricular zones that remain abundant in the CNS, generating myelinating oligodendrocytes throughout adult life [5]. Therefore, if the remyelination is sufficient, the loss of function experienced by people with multiple sclerosis (pwMS) may be transient [6].

Over the last two decades, pathology-supported genetic–testing (PSGT) research conducted at our institution concluded from published literature and our own results [6–11] that risk factors for oligodendrocyte demise and subsequent MS diagnosis and progression fall into two categories: (1) *deficiencies* and (2) *aggravators* (Table 1).

Nutrition is widely recognised as a potential environmental factor contributing to the development of MS, but its role as complimentary treatment remains unclear and has been largely overlooked. Nevertheless, interest in nutrition's impact on MS has grown since the 1950s, when Dr Roy Swank's studies suggested a link between dietary saturated fat intake and MS prevalence and disability [22]. Although Swank's findings were observational and not based on randomized controlled trials (RCTs), a recent Systematic Review and Network Meta-analysis of Randomized Trials by Snetselaar et al. has addressed this limitation [28]. The analysis included 12 RCTs comparing eight dietary interventions: low-fat, Mediterranean, ketogenic, anti-inflammatory, Paleolithic, fasting, calorie restriction, and control (usual diet) [28]. Notably, the Wahls–modified Paleolithic and Swank low-fat diets both demonstrated significant improvements in quality of life (QOL) and reduced fatigue [21], while the Mediterranean diet was associated with significantly lower disability in pwMS [29]. A very-low-fat, plant–based diet RCT, which excluded both saturated fat and unsaturated oils including olive oil, found an improvement in fatigue and QOL but not in disability or MRI outcome measures [30]. In contrast, WML volumes decreased significantly in some brain regions in pwMS who followed an anti-inflammatory diet [31].

A limitation of the published diets for MS is that their benefits for preventing disability progression may be negated by other risk factors, such as smoking (active or passive) or lack of exercise. These factors can impair the cerebral circulation which is vital for the adequate delivery of oxygen and nutrients to the oligodendrocytes [23, 24]. Not all pwMS are affected similarly; therefore a PSGT Program was developed at our Institution to identify the risk factor(s) relevant for specific individuals by assessing biochemical, genetic, diet and lifestyle information. The results are compiled into adaptable reports, enabling healthcare providers to offer personalized recommendations based on the risk factors identified, such as biochemical imbalances revealed in the blood test results and lifestyle modification [6].

The present proof-of-concept pilot study tested the hypothesis that relapse-associated worsening (RAW) and WML accumulation could be delayed, and serious MS disease could be prevented, by addressing all risk factors contributing to oligodendrocyte damage. The study used 3-Tesla MRI (3-T MRI) to measure WML volumes.

## 2. Methods

### 2.1. Study Participants, MRI Procedures, and Software Analysis

This study formed part of a case-control substudy that recruited 51 pwMS (48 females and 3 males) and 25 controls without neurological symptoms, for vascular ultrasound of the carotid arteries [23] and assessment of disability using the Expanded Disability Status Scale (EDSS) [32], as well as genetic studies [6]. Twenty-five of these female pwMS as well as 25 age-matched female controls who had demographic, biochemical, and lifestyle data were randomly recruited for 3-T MRI at the Cape University Body Imaging Centre (CUBIC), University of Cape Town.

Inclusion criteria were females, ages 18–60 years with neurologically confirmed MS according to the McDonald 2001 criteria [33], MRI compatible, nonsmokers, and normotensive (including hypertension managed medically). Exclusion criteria were other neurological diseases (e.g., neuromyelitis optica, acute disseminated encephalomyelitis, meningitis, or epilepsy), any factors contrary to having an MRI scan (e.g., mechanical devices and metal fragments), smoking or smoking–related medical pathologies such as chronic obstructive pulmonary disease, uncontrolled hypertension, diabetes, or previous cardiac/neurological conditions. Controls without neurological disorders were age and sex matched.

Of the recruited participants, 22 pwMS and 21 controls completed the MRI. Demographic data recorded were age at MRI, disease duration (years with MS), MS subtype, and medication. Disease-modifying treatment (DMT) was interferon-*β* in 10 of the pwMS. The pwMS voluntarily decided whether to implement the recommendations to follow the PSGT Program or not. Of the 22 pwMS, 13 had followed the Program for more than 10 years, while 9 had not followed the program.

As the sample size calculated was 25 MS and 25 controls as previously reported [6], this was the number of pwMS and controls randomly recruited from the vascular ultrasound study [23]; however, 7 of these (3 pwMS and 4 controls) could not have an MRI due to concerns of claustrophobia or illness. In total, 22 females with MS and 21 female controls signed consent to be included in the 3-T MRI assessment.

Participant preparation occurred outside of the scan room, and this process included an explanation of the procedure to ensure that the participant was comfortable as well as a CUBIC MRI compatibility checklist, signed by both the participant and the radiographer, before removing their clothing, jewellery, etc., and putting on a cotton gown. Scanning proceeded in the head-first, supine position, and a wedge was placed under the knees of each participant to lessen their discomfort during the scan. To ensure privacy, participants were covered with a sheet or a light blanket. A panic ball was handed to all participants to be used as an alarm in case they required any assistance during the procedure. MRI-compatible headphones were placed over their ears to reduce the acoustic noise of the scanner and to ensure participants could hear instructions during the scan.

Images were acquired on a 3-T Siemens Magnetom Skyra scanner using a standard 32-channel transmit–receive (RF) head coil. The protocol included the following sequences: fluid attenuation recovery (FLAIR) in axial and sagittal planes, proton density (PD), T2 (in coronal plane), magnetization prepared rapid gradient echo (MPRAGE), susceptibility weighted imaging (SWI), diffusion tensor imaging (DTI), and readout segmentation of long variable echo-trains (RESOLVE) images. The axial FLAIR and T1 sequences were specifically employed for the WML volume data analyses to provide contrast and expose pathology. Imaging parameters for these sequences are shown in Table 2.

Lesion volumes were determined using FLAIR and T1 images with Sequence Adaptive Multimodal SEGmentation (SAMSEG) software [34], part of FreeSurfer 7.2. In this study, we utilized the freely available software package SAMSEG [35] to quantify WMLs from MRI data. SAMSEG takes multicontrast MRI data as input, regardless of scanner or pulse sequence used, and segments 41 brain structures as well as WMLs using a generative model [34]. We used both T1 and FLAIR images as input, as SAMSEG benefits from the T1 contrast for segmenting cortical gray–white matter boundaries and from the FLAIR contrast for optimal delineation of lesions. Apart from coregistering the FLAIR and T1 images and reslicing them to the same resolution, SAMSEG requires no image preprocessing. Although manual labelling has been shown to be slightly more accurate [34], automated methods eliminate intra and inter-rater discrepancies. Automated tools that can quickly and reliably characterise the shape and size of WMLs are of great value for tracking disease progression and evaluating treatment efficacy from MRI data.

### 2.2. Diagnosis and Disability Assessments

PwMS were diagnosed according to the McDonald criteria (2001) before entering the study [33]. All pwMS had 1.5-T MRIs at diagnosis and follow-up, which were evaluated by their respective neurologists. Our study neurologist carefully reviewed the neurology and radiological reports of the pwMS included to confirm the original diagnosis, considering factors such as WML position, oligoclonal bands, and elevated IGG-Index, to ensure adherence to the McDonald criteria [33]. Disability was assessed and quantified by a clinician using the EDSS. This widely used scale monitors changes in disability levels ranging from 0 to 10 in 0.5 unit increments, with higher scores indicating greater disability [32].

### 2.3. The PSGT Program

Assessment of the personalized risk factors and optimization of cerebral nutrients in pwMS were performed at our Institution in a PSGT Program as described previously [6, 8, 9]. Briefly, participants completed a lifestyle questionnaire to provide demographic data and information on family and personal medical history, alcohol intake, smoking, and physical activity. Biochemical tests were performed to identify possible deficiencies of iron, vitamin D and vitamin B12, or excess cholesterol/homocysteine and increased inflammation (C-reactive protein (CRP). Genetic testing was performed to identify clinically relevant variants in metabolic pathways. All data were integrated to generate adaptable personalized reports [6], enabling clinicians to address imbalances revealed in the blood test investigations and to mitigate all the risk factors through nutritional supplementation and modification of lifestyle. The pwMS were followed up regularly to ensure that they followed the Program, while new results were recorded and compared with previous data captured on a secure database (http://www.gknowmix.com/). A mobile phone app has subsequently been developed to enable online consent to complete the questionnaire [36]. The pwMS were also encouraged to contact the Genetic Care Centre if they had any questions or experienced symptom flare-ups. However, the pwMS were free to choose not to follow the program, and some of them opted to use DMTs instead.

### 2.4. Biochemical Analysis

Blood was drawn for biochemistry testing between 09:00 and 10:30 to standardize for diurnal variation. Biochemical analysis of the following biomarkers was performed by an accredited pathology laboratory: iron parameters, ferritin, CRP, total cholesterol, homocysteine, serum folate, vitamin B12, and 25-OH vitamin D as reported previously [11].

### 2.5. Genetic Testing

All participants underwent genetic testing as published previously [6] to identify SNPs in the following metabolic pathways: lipid and lipoprotein metabolism, homocysteine and folate metabolism [11]; haemostasis and thrombophilia; iron overload and iron deficiency [16]; hypertension [11]; obesity and insulin resistance [6]; and inflammation, detoxification, and oxidative stress [20]. P4 medicine (participatory, personalized, predictive, and preventive medicine) was enabled by genetic and environmental factors aligned with biochemical results influencing clinically relevant metabolic pathways [6].

### 2.6. Statistical Analysis

Statistical analyses were performed using Statistica Version 13.4.0.14. Differences in lesion volumes and EDSS parameters between study groups were tested using one-way analysis of variance. Normality was assessed by inspecting normal probability plots which were, in all cases, found to be acceptable. Levene's test was done to test for homogeneity of variance and was in all cases accepted. In cases where it did not hold, the Welch test was also done. However, the outcome of the Welch test was the same as for the *F*-test of the ANOVA, so only the ANOVA result was reported. Pearson's correlations were reported to test for relationships between lesion volumes, lifestyle parameters, and EDSS. Effect sizes of the ANOVA results were calculated using the Hedges *D*-test.

A 5% significance level (*p* < 0.05) was used as a guideline for statistical significance.

### 2.7. Ethics

This study was a collaborative investigation between Stellenbosch University (SU), the University of Cape Town (UCT), and the Cape Peninsula University of Technology (CPUT) in the Western Cape, South Africa. Ethics approval was granted by the Faculty of Medicine and Health Sciences Research Ethics Committee of the SU (references N07/09/203 and N09/08/224) and the Faculty of Health and Wellness Sciences Research Ethics Committee of CPUT (reference no: CPUT/HW-REC 2019/H27). As a postgraduate project, the study was conducted according to the code of ethics of the World Medical Association, in accordance with the Declaration of Helsinki (1964) [37]. All study participants gave signed informed consent for the MRI and for their data to be used for a research publication.

## 3. Results

Data generated characterized lesion load, disability, demographic, and biochemical markers. Clinical data of 22 pwMS and 21 controls are summarized in Table 3.

### 3.1. MRI Results of pwMS and Controls

Mean lesion volumes (mm^3^) were significantly greater (*p* < 0.001) in pwMS (10,262 ± 10,294) than in controls (768 ± 1001), that is, the mean WML volumes of pwMS were 10.3 mL versus 0.76 mL in the controls. None of the controls had neurological symptoms; however, SAMSEG software identified WMLs in some control participants. Radiologist reports confirmed the presence of age-appropriate white matter hyperintensities that were identified as WMLs by the software. There was a trend for a negative association between WM hyperintensities and vitamin D concentrations in the controls (*r* = −0.44; *p* = 0.058). Alcohol intake was not significantly associated with WML volumes, either in pwMS or controls (*r* = −0.27, *p* = 0.22 and *r* = −0.32, *p* = 0.18, respectively). None of the genetic variants tested were associated with WML volumes, nor was physical activity.

In the pwMS, associations were assessed between WML volumes and age, disease duration (years with MS), and disability (EDSS). Of the 22 pwMS, 10 were on DMT, all of whom were prescribed Interferon-*β*. WML volumes were associated significantly with the EDSS (*p* < 0.02), but not with age, disease duration, or DMT use (Table 4).

**Figure 2:**
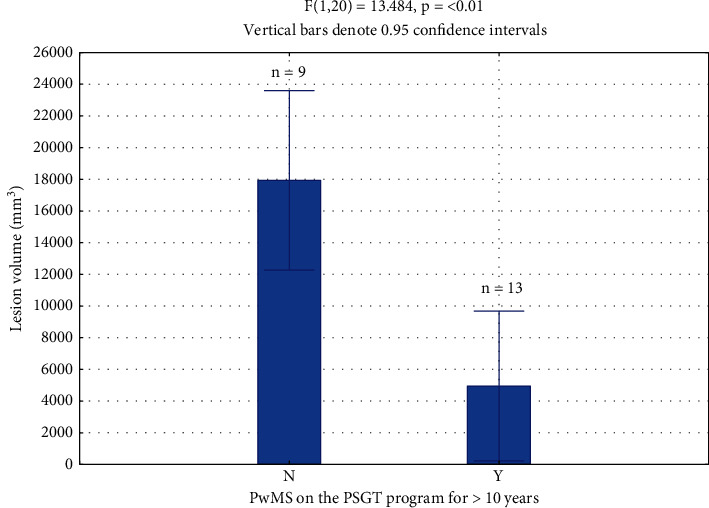
Significant difference between white matter lesion volumes of pwMS (*n* = 13) who had followed the PSGT Program for >10 years and pwMS (*n* = 9) who had not, 4950 ± 5303 versus 17934 ± 11139 mm^3^ (*p* = 0.002) Hedges *D* = 1.53 (large effect size). N = no; Y = yes.

Of the 22 pwMS, 13 had followed the PSGT program for more than 10 years, while 9 had not followed the program. The pwMS who had followed the program had significantly smaller WML volumes (4950 ± 5303 mm^3^) than the pwMS not on the program (17,934 ± 11,139 mm^3^; *p* = 0.002) Hedges *D* = 1.53 (large effect size). (Figure 2).

The EDSS values in the pwMS who had followed the PSGT Program were significantly (*p* = 0.02) lower (mean 2.0, range 1–3.5) than the pwMS who had not (mean 4.2, range 1–8; Hedges *D* = 1.08 [large effect size]). The pwMS on the program had EDSS values in the minimal to mild range [32] after more than 10 years, being freely ambulatory; nine of them had cognitive-intensive employment positions: two teachers, a musician (lounge pianist), a lecturer, a chartered accountant, a medical doctor, a web developer, an owner of a social media company, and an administrator/bookkeeper. Four were retired/unemployed. None of the 13 pwMS on the Program had transitioned to secondary progressive MS (SPMS). Of the pwMS who had a disease duration of more than 10 years and had not followed the program (*n* = 4), two had to use a cane for walking, and 2 were wheelchair bound. Three of the four had transitioned to SPMS. The other 5 pwMS not following the program had a disease duration of less than 10 years.

**Figure 3:**
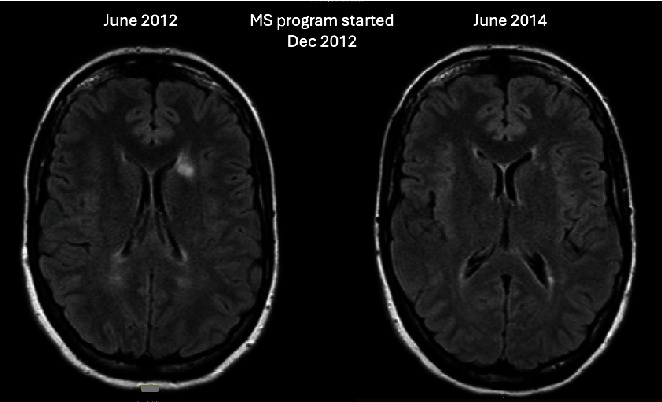
Resolution of white matter lesions in one of the designated pwMS who could not be scanned using 3-T MRI due to claustrophobia. MRIs in 2012 and 2014 were done in a hospital using sedation under clinical supervision by a neurologist.

Figure 3 shows the 1.5-T MRIs of a female diagnosed with MS in 2007, who was invited to be included in the 3-T MRI study but was unable to comply due to claustrophobia. She had MRIs in June 2012 and again in June 2014 in hospital under sedation with clinical supervision by her neurologist, who treated her with Avonex and also referred her to the PSGT program (2012). According to the neurologist's report, the comparison between the MRIs dated June 2012 and June 2014 showed that the high signal intensity foci on FLAIR and T2 had all decreased in size and signal intensity. The minimal high signal in the right temporal lobe had also decreased. In addition, there were no new enhancing areas within the brain. Her EDSS in 2012 was 2.0, and when reassessed in 2014, it had improved to 1.5. At the time of the present study (2019), her EDSS was holding steady at 1.5.

The MRIs of another participant in the present study also showed smaller WMLs at follow-up (2009) after entering the PSGT Program in 2007. At diagnosis in 2006, the radiologist's report noted: “FLAIR and T2 sequence hyperintensities which are perpendicularly orientated in relation to the ventricle (Dawsons' fingers), as well as a lesion in the pons”. In contrast, the 2009 follow-up MRI report stated: “The previous pontine lesion has resolved without residual signal changes and there is only slight scarring at the site of the previous right periventricular demyelinating lesions. No new or progressive lesions”. This was confirmed in the present 3-T MRI study, at which time her EDSS was 1.5. Notably, at diagnosis, her iron levels were extremely low, prompting her neurologist to administer intravenous iron. She requires a daily iron intake of 10 mg to prevent deficiency. Her favorable disease status has enabled her to maintain an active lifestyle, including running half-marathons.

## 4. Discussion

The present study revealed that the pwMS who followed the PSGT Program for more than 10 years had WML volumes three times smaller than those who did not follow the program, a significant difference despite the small study sample. Notably, WML volumes were significantly associated with disability but not with age, disease duration, or DMT use. The smaller WMLs could be attributed to a pause in their accumulation or partial resolution, as demonstrated in the two case examples above. We previously published a case series of pwMS who had followed our program and achieved favourable disability outcomes after more than 10–20 years; one became an ultramarathon athlete, and the other experienced a reversal of disability from EDSS 7.5–2.0, both maintaining an adequate intake of iron [6]. The original diagnoses of these pwMS have been verified [6].

The present study results tentatively suggest that mitigating MS risk factors may alleviate “smouldering MS,” the chronic inflammation underlying “real MS” [38], characterized by relentless autoimmunity-driven disease worsening. Instead, we observed remission in the pwMS following the PSGT program. Further studies are needed to confirm whether this supports the findings of an international Colloquium convened in 2007, where the apparent two-way relationship between cell damage and autoimmunity was discussed [39]. The Colloquium identified five etiologic and pathophysiologic processes that may influence the initiation, development, and perpetuation of autoimmunity: inflammation, infection, apoptosis, environmental exposure, and genetics. Notably, excessive apoptosis overwhelmed the monocyte-macrophage system, leading to autoimmunity, while conversely, reducing oxidative damage and inflammation may restore immunologic balance and induce remission [39]. Neuropathological studies have suggested that the chronic inflammation underlying “smouldering MS” may be caused by the cytokines and oxidative molecules released by activated microglia/macrophages when they scavenge apoptotic oligodendrocytes and dysfunctional myelin [1, 2]. The PSGT program for MS was developed with these insights in mind.

The rationale for including specific risk factors in the program was informed by the literature and our own research experience, particularly regarding deficiencies of iron and vitamin B12-folate methylation metabolism which play a crucial role in mitochondrial energy production [40]. Oligodendrocytes are extremely energy dependent due to their responsibility to produce and maintain a vast amount of myelin, including cholesterol that is not obtained from the circulation but has to be synthesized by the oligodendrocytes themselves [41]. Adequate iron must be transported into the mitochondria to support this excessive energy generation [8, 16]. If the oligodendrocytes experience energy deficits, they perish [8, 41–43]. Furthermore, oligodendrocytes require optimal folate-vitamin B12 metabolism to methylate myelin basic protein (MBP) during myelin synthesis. Incomplete methylation of MBP compromises myelin integrity [44] and may perpetuate chronic inflammation. Vitamin B12 is a cog in the wheel of methylation metabolism and its deficiency leads to demyelinating neurological conditions [45, 46].

Vitamin D deficiency is a causal risk factor for MS [12, 47] as it plays a crucial role in modulating the activated immune system in MS [48]. Serum concentrations of vitamin D are associated with sunlight exposure. According to the Atlas of MS of the MS International Federation (2023), the highest MS prevalence globally is in the Northern countries such as Canada (290 cases per 100 000), whereas South America has fewer cases, and the lowest prevalence is in Sub-Saharan Africa; with Tanzania reporting only 0.01 cases per 100 000 (just 6 recorded cases) [49]. Epstein–Barr virus infection has also been identified as a causal factor for MS [12]. Adequate vitamin D intake, potentially up to 20,000 IU/day for short time periods [50], may offer protection against this risk. Optimal serum values for vitamin D in MS are suggested to be 30–100 nl/mL (75–250 nmol/L) [48].

Oxidation and inflammation are extremely prevalent in MS and need to be constantly counteracted by adequate intake of antioxidants; daily intake of more than five fruits/vegetables is significantly inversely associated with disability [11]. OPCs are particularly vulnerable to oxidation because of a lack of intrinsic antioxidant capacity [51]. If OPCs are subjected to the same risk factors as the oligodendrocytes that they are meant to replace, they may also not survive. Environmental toxins such as smoking and pesticides aggravate relapses and disability [6, 11, 52]. Avoiding food allergens reduces fatigue and improves QOL in pwMS who are susceptible to these food sensitivities [21].

Furthermore, unsaturated oils (olive oil, Omega-3, and evening primrose oil) also provide protection against inflammation, confirmed by a study that showed an association between MS disability and a genetic variation in *FABP2*, that codes for a transporter of fatty acids [6]. Saturated fat intake, which contributes to obesity, was the first environmental causative risk factor for MS to be identified by Swank and Grimsgaard [22]. Swank and Goodwin postulated that saturated fat impedes the nourishment of tissues due to rigid chylomicron aggregates blocking the small blood vessels in the CNS, in contrast to smaller, less rigid aggregates formed from unsaturated oils in the diet [53]. A study of the Mediterranean diet in MS [54] found that higher consumption of olive oil, nuts, and wine and lower consumption of pastries and sugary beverages were associated with less disability, while depression was worse among people who ate butter, drank sugary beverages, and ate less fish. Participants in our program have stated that stress is an aggravating risk factor for MS and MS relapses (Gknowmix questionnaire entries, unpublished results) while exercise improves blood flow and alleviates stress and pain [6].

### 4.1. Limitations

The study's greatest limitations are its small sample size, observational nature, and the current lack of RCTs. Another limitation is that 3-T MRIs and software for measuring WML volumes were not available at baseline for the participants in this study 10 years ago; therefore WML volumes could only be measured at present. Future studies should prospectively follow up pwMS to assess the progression or cessation of WML accumulation when risk factors for MS are mitigated. Additionally, when the pwMS were diagnosed 10 years ago, antibody tests were not part of the diagnostic criteria as they are now; therefore, the McDonald criteria were applied as published at the time [33]. Antibody tests were later conducted on some but not all pwMS. However, since serum anti-CNS autoantibodies specific for MS have not yet been identified [55], antibody tests are primarily useful when the clinical presentation is at variance with a diagnosis of MS. Furthermore, a limiting factor in measuring WML volume is the need to specify a probability threshold for lesion identification in automated methods. If the threshold is too low, it may result in false positives, while a too high threshold may reduce sensitivity. Since our data were acquired with a standardized protocol on the same scanner, a suboptimal threshold selection would lead to a systematic over- or underestimation of lesion volume across subjects.

The strength of this study lies in the significantly smaller WML volumes and favourable disease outcomes in pwMS who followed the PSGT program over 10 years, compared to those who did not (Figure 2).

## 5. Conclusion

This pilot case-controlled study supports the hypothesis that targeted dietary, nutritional, and lifestyle interventions may enhance brain health and neural processes. The current study serves as a necessary first step in understanding these processes in MS; highlighting the need for future research, including RCTs, to determine whether the PSGT program can delay WML formation and prevent severe disease by mitigating MS risk factors, optimizing nutrition, and ensuring adequate blood flow to the CNS.

## Figures and Tables

**Table 1:** Genetic, metabolic, and environmental risk factors for disability progression in MS.

**Deficiencies**	**Aggravators**
Vitamin D deficiency [12], enhanced by *VDR* genetic variations [13, 14]	Oxidation and inflammation [8, 15]
Iron deficiency, enhanced by *TMPRSS6, TF, SLC25A37*, and *CUBN* genetic variations [7, 10, 16]	Smoking, active or passive [11, 17]
Vitamin B12/folate deficiency, enhanced by *MTHFR* genetic variations and high homocysteine [11, 18–20]	Chronic infections such as EBV [12]
Deficiency of antioxidants (fruit/vegetables) in the diet [11]	Chronic allergies or food sensitivity [21]
Deficiency of unsaturated oils in the diet, enhanced by the *FABP2* rs1799883 genetic variant [6]	High dietary saturated fat [22], which causes increased cholesterol and obesity, enhanced by *FTO* genetic variations [11]
Oxygen/nutrient deficits in the CNS due to increased vascular intimamedia thickness [23, 24] and decreased blood flow in areas of the CNS	Environmental toxins [25] including inhalation of poisons sprayed for insects in the home or garden [6]
Lack of exercise/sedentary lifestyle [26]	Psychological stress [27]

**Table 2:** MRI sequences and parameters.

**MRI sequences**	**Repetition time (ms)**	**Echo time (ms)**	**Slice thickness (mm)**	**Field of view**	**Matrix %**	**Distance factor %**
FLAIR (axial)	9400.0	78.0	3.0	256 mm	256 × 100	0
T1w MPRAGE (axial)	1720.0	2.47	2.0	100%	256 × 100	50

Abbreviations: FLAIR = fluid–attenuated inversion recovery, T1w MPRAGE = magnetization prepared rapid gradient echo.

**Table 3:** Clinical data of 22 pwMS and 21 controls who had 3-T MRI assessments.

**Parameter**	**PwMS on PSGT program for > 10 years**		**All pwMS (** **n** ** = 22)**	**Controls (** **n** ** = 21)**
	**Yes**	**No**		
Females *n*	13	9	22	21
Age at MRI: years, mean (range)	52 (36–65)	48.8 (23–71)	50.7 (23–71)	48.3 (25–72)
Disease duration: years, mean (range)	17.5 (11–29)	12.2 (1–27)	15.4 (1–29)	NA
MS subtype: RR	13	6	19	NA
MS subtype: SP	0	3	3	NA
Disease-modifying therapy	3	7	10	NA
Untreated *n* (%)	10	2	12	NA
BMI kg/m^2^, mean (range)	25.2 (21.5–35)	25.5 (19–32)	25.3 (19–35)	27.7 (21.4–43)
Cholesterol mmol/L, mean (range)	5.5 (3.9–8)	5.4 (4.4–6.8)	5.4 (3.9–8)	5.6 (3.9–7.9)
Vitamin D (ng/mL), mean (range)	28.8 (9.2–63)	33.2 (13.3–63.9)	30.6 (9.2–63.9)	27.4 (8.4–78.7)
Smokers *n*	0	0	0	0
Previous smokers *n*	4	2	6	2
Passive smokers *n*	4	5	9	7
Fruits/veg > 5: days/week, mean (range)	5 (0–7)	4.4 (0–7)	4.8 (0–7)	3.9 (0–7)
EDSS, median (range)	**2 (1–3.5)**	**4.2 (1–8)**	2.9 (1–8)	NA

*Note:* Bold indicates values with significant difference (*p* = 0.02).

Abbreviations: Fruits/veg > 5: days/week = more than five fruits and/or vegetables: days per week, NA = not applicable, RR = relapsing-remitting, SP = secondary progressive.

**Table 4:** Associations of WML volumes in pwMS with age, disease duration, disability, and medication use.

**Variable**	**Pearson correlation**	**p** ** value**
Age at MRI	0.21	0.350
Disease duration	0.08	0.709
EDSS	0.73	**< 0.02**
DMT use		0.70^a^

*Note:* Bold indicates values with significance.

^a^ANOVA test for equal means.

## Data Availability

The data that support the findings of this study are available on request from the corresponding author. The data are not publicly available due to privacy or ethical restrictions.
